# A triton X-100 assisted PMAxx-qPCR assay for rapid assessment of infectious African swine fever virus

**DOI:** 10.3389/fmicb.2022.1062544

**Published:** 2022-12-05

**Authors:** Huan Liu, Fei Meng, Raphael Nyaruaba, Ping He, Wei Hong, Mengwei Jiang, Dongqing Liu, Wenhao Zhou, Dan Bai, Junping Yu, Hongping Wei

**Affiliations:** ^1^CAS Key Laboratory of Special Pathogens and Biosafety, Center for Biosafety Mega-Science, Wuhan Institute of Virology, Chinese Academy of Sciences, Wuhan, China; ^2^College of Life Sciences, University of Chinese Academy of Sciences, Beijing, China; ^3^African Swine Fever Regional Laboratory of China (Wuhan), Wuhan, China; ^4^Comprehensive Agricultural Law Enforcement Bureau, Wuhan, China

**Keywords:** African swine fever virus, infection, disinfectant, propidium monoazide, TritonX-100, quantitative PCR

## Abstract

**Introduction:**

African Swine Fever (ASF) is a highly infectious disease of pigs, caused by *African swine fever virus* (ASFV). The lack of vaccines and drugs makes strict disinfection practices to be one of the main measurements to curb the transmission of ASF. Therefore, it is important to assess if all viruses are inactivated after disinfection or after long time exposure in their natural conditions. Currently, the infectivity of ASFV is determined by virus isolation and culture in a biosafety level 3 (BSL-3) laboratory. However, BSL-3 laboratories are not readily available, need skilled expertise and may be time consuming.

**Methods:**

In this study, a Triton X-100 assisted PMAxx-qPCR method was developed for rapid assessment of infectious ASFV in samples. PMAxx, an improved version of propidium monoazide (PMA), can covalently cross-link with naked ASFV-DNA or DNA inside inactivated ASFV virions under assistance of 0.1% (v/v) TritonX-100, but not with ASFV-DNA inside live virions. Formation of PMAxx-DNA conjugates prevents PCR amplification, leaving only infectious virions to be detected. Under optimum conditions, the limit of detection of the PMAxx-qPCR assay was 2.32log_10_HAD_50_/mL of infectious ASFV. Testing different samples showed that the PMAxx-qPCR assay was effective to evaluate intact ASFV virions after treatment by heat or chemical disinfectants and in simulated samples such as swine tissue homogenate, swine saliva swabs, and environmental swabs. However, whole-blood and saliva need to be diluted before testing because they may inhibit the PCR reaction or the cross-linking of PMAxx with DNA.

**Conclusion:**

The Triton X-100 assisted PMAxx-qPCR assay took less than 3 h from sample to result, offering an easier and faster way for assessing infectious ASFV in samples from places like pig farms and pork markets.

## Introduction

African Swine Fever (ASF) is a highly contagious and epidemic disease of pigs caused by a large, icosahedral, enveloped, double-stranded DNA virus named *African swine fever virus* (ASFV), which is the sole member of the family *Asfarviridae* (Galindo and Alonso, 2017; Dixon et al., 2019). Different isolates of ASFV exhibit variable virulence (Portugal et al., 2015). ASFV in blood (Plowright and Parker, 1967), feces (Fischer et al., 2020a), urine (Davies et al., 2017) and tissues (Mazur-Panasiuk and Wozniakowski, 2020) can survive in different environments for long time periods. Contaminated animal feed, pork, clothing, footwear, farming tools, equipment and vehicles etc. will increase the risk of ASFV transmission. Therefore, in the absence of commercial vaccines and therapeutic agents against ASFV (Teklue et al., 2020), culling infected pigs and strict disinfections are the main measurements for protecting the pig industry. It is therefore important to assess whether there exists infectious ASFV after disinfection.

The gold standard for evaluating the infectivity of ASFV after disinfection is virus isolation and culture. However, this method has some shortcomings: (1) A biosafety level 3 (BSL-3) laboratory and porcine primary macrophage cells are needed for ASFV isolation and culture, which are expensive and not available to standard microbiology labs; (2) It takes at least 5 days to determine infectivity; (3) Different sample pretreatments are needed to remove cell toxicity of the chemical disinfectants before virus culture; and (4) well-trained personnel are needed to perform infectivity tests. Due to these strict and unfavorable conditions, rapid and regular monitoring of infectious ASFV is limited, especially for low resource settings.

Real time or conventional PCR assays are recommended by the World Organization for Animal Health (WOAH) for rapid screening and diagnosis of ASFV (Wang et al., 2020). However, these assays cannot determine viral infectivity of ASFV. Some viability dyes such as ethidium monoazide (EMA; Elizaquivel et al., 2014), propidium monoazide (PMA; Sarmento et al., 2020), and an advanced version of PMA dye, PMAxx (Shirasaki et al., 2020), can penetrate damaged or destroyed viral capsids but not intact capsid (Lee et al., 2018) and intercalate covalently into the chains of the nucleic acid after photoactivation to prevent the PCR amplification of these nucleic acids (Li et al., 2015; Nyaruaba et al., 2022). These viability dyes combined with real time PCR (qPCR), have been successfully applied to discriminate infectious viruses from inactivated ones in various studies involving Hepatitis A virus (HAV; Randazzo et al., 2018), Hepatitis E virus (HEV; Schielke et al., 2011), Human rotaviruses (HuRV; Coudray-Meunier et al., 2013), Human Norovirus (HuNoV; Razafimahefa et al., 2021), Porcine epidemic diarrhea coronavirus (PEDV; Puente et al., 2020), and the severe acute respiratory syndrome coronavirus 2 (SARS-CoV-2; Canh et al., 2021). Compared to virus culture, the qPCR-based viability assays overcome the requirements of a BSL-3 laboratory and cell culture for assessing viral infectivity. Additionally, the qPCR technology is widely and readily available even to low resource settings, making it an attractive option to the conventional culture technique.

In this study, we aimed to develop a qPCR assay combined with PMAxx pretreatment for rapid assessment of infectious ASFV in different bio-matrixes after chemical inactivation or heat-treatment. The technique is simple, fast, and can be easily adapted by normal molecular diagnostic laboratories to monitor infectious ASFV.

## Materials and methods

### Virus stocks and cell culture

Porcine alveolar macrophages (PAMs) were prepared from bronchoalveolar lavage and maintained in Roswell Park Memorial Institute (RPMI) 1640 medium (Gibco, United States) supplemented with 10% fetal bovine serum (FBS, Sigma, United States), 100 U/ml penicillin, 100 μg/ml streptomycin and 250 ng/ml amphotericin B (Beyotime Biotechnology, China) at 37°C with 5% CO_2_. ASFV (CSTR: 16533.06. IVCAS 6.7494, genotype II) was stored at −80°C in the biosafety level 3 (BSL-3) facility of Wuhan Institute of Virology, Chinese Academy of Sciences (WIV-CAS). All the experiments involving infectious ASFV were performed in the BSL-3 laboratory. The titer of ASFV stocks were determined by the hemadsorbing (HAD) test. Briefly, 4 × 10^4^ cells/well of PAMs were seeded into 96-well plates and infected with 10-fold diluted ASFVs. After 1-day infection, 1% porcine erythrocyte cell suspensions stored in PBS (Gibco, United States) were added into each well. The phenomena of hemadsorption were observed over 7 days by a microscope. The 50% hemadsorbing dose (HAD_50_) was calculated by the Reed and Muench method (Zhao et al., 2019).

### Reagents used and sources

Reagents used to develop the assays and perform viability experiments were purchased from different companies. In summary, the PMAxx (40,069, 20 mM in H_2_O) was purchased from Biotium (United States), TritonX-100 from Sigma-Aldrich (United States), Virkon™ S from DuPont (United States), and Disinfectant Basi containing 4.0–4.99% (w/v) chlorine from Yiheng (Dezhou, China). Primers and probes were synthesized by Sangon Biotech (Shanghai, China). All other chemical reagents used in the experiments were purchased from Sinopharm (Shanghai, China) except otherwise stated. Double distilled water was used in all experiments.

### Virus inactivation and sample preparation

#### Heat inactivation of ASFV

A series of 10-fold gradient dilutions of ASFV suspensions were prepared by diluting the ASFV stock solution with phosphate buffer solution (PBS, pH 7.4). Parts of the dilution series were aliquoted and inactivated at different temperatures (60°C, 70°C, and 95°C) for 20 min, respectively. After heat treatment, all the aliquots were centrifuged at 10,000 × g (4°C) for 5 min to obtain the supernatants which were then collected and stored on ice until use. Each step was performed in triplicate.

#### ASFV disinfection by chemicals

The chemical disinfectants and reaction conditions used in this study are summarized in Table 1. These chemicals were verified as ASFV disinfectants in previous studies (Krug et al., 2018; Juszkiewicz et al., 2019, 2020; McCleary et al., 2021). Briefly, aliquots of 180 μl ASFV suspensions with 4.3log_10_HAD_50_/mL were mixed with either 20 μl of commercially purchased 84 surfactant [composed of sodium hypochlorite (NaClO) with the chloride concentration between 4 and 4.99% (w/v)], 25% (w/v) glutaraldehyde (GA), acetic acid (HAc), 8% (w/v) sodium hydroxide (NaOH), or 10% (w/v) Virkon (VK), respectively. After incubation at room temperature for 30 min, the disinfection was stopped by immediately adding the corresponding neutralizer and PBS to a total volume of 1 ml. NaOH and HAc were neutralized by 0.2 M hydrogen chloride (HCl) and 0.2 M NaOH, respectively. 7% (w/v) glycine was used to stop the reaction of glutaraldehyde (Cheung and Brown, 1982). The neutralizer used for the NaClO and VK was 0.5% (w/v) sodium thiosulphate (Na_2_S_2_O_3_; Olmez-Hanci et al., 2014; Sahebi et al., 2020). Finally, all the disinfected samples were centrifuged at 10,000 × g (4°C) for 5 min to get the supernatants prior to storage on ice until use. All the treatments were performed in triplicate.

**Table 1:** Chemicals and corresponding neutralizers used for ASFV disinfection.

Chemicals	Disinfection conditions			Neutralizer
	Method	Time (min)	Temperature (°C)	
0.4–0.499% (w/v) NaClO	Immersion	30	22–25	0.5% (w/v) Na_2_S_2_O_3_
2.5%(w/v) GA	Immersion	30	22–25	7% (w/v) glycine
10% (v/v) HAc	Immersion	30	22–25	0.2 M NaOH
0.8% (w/v) NaOH	Immersion	30	22–25	0.2 M HCl
1% (w/v) VK	Immersion	30	22–25	0.5% (w/v) Na_2_S_2_O_3_

### Optimization of PMAxx and triton X-100 pretreatment

**Figure 1:**
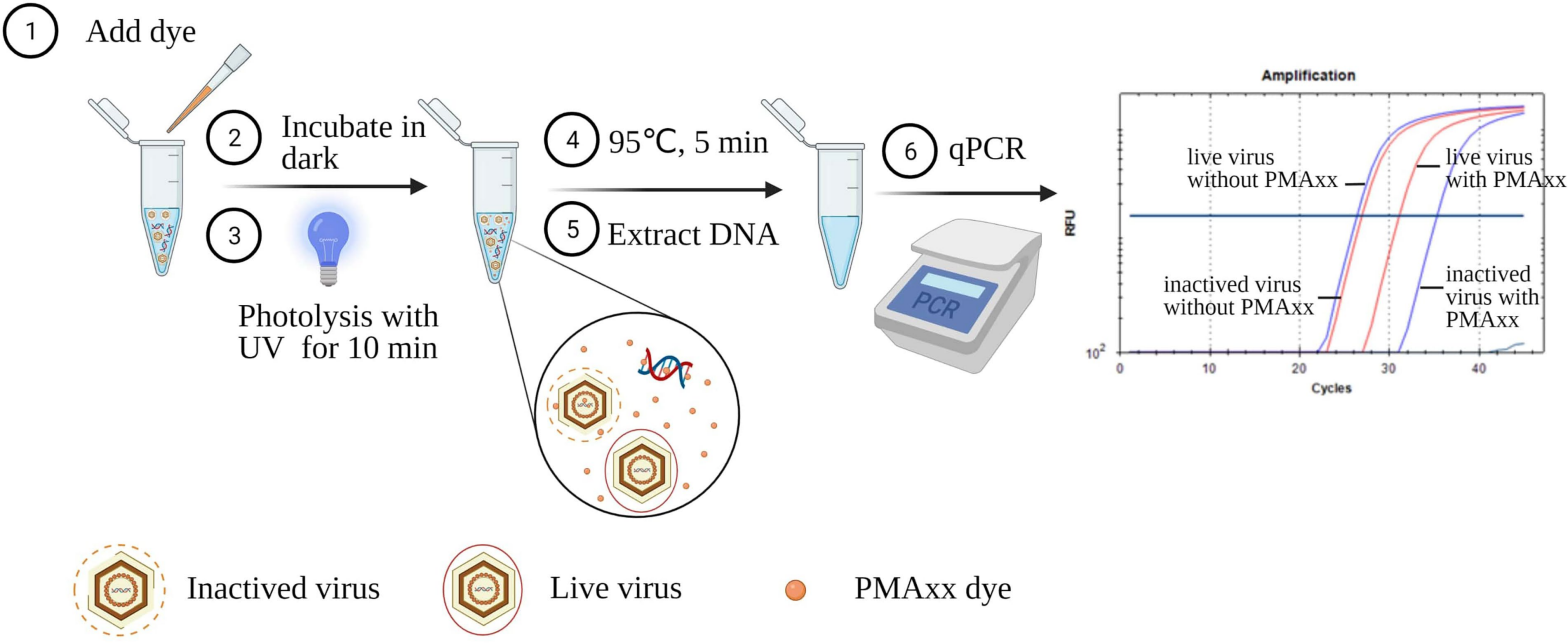
Schematic diagram of the PMAxx-qPCR assay for discriminating infectious and inactivated ASFV. The schematic was produced on https://biorender.com.

Extracted ASFV DNA using the Blood viral DNA extraction kit (Qiagen, catalog 51104) and virus suspensions before and after inactivation at different conditions were used to optimize the conditions of the PMAxx-qPCR assay. As shown in Figure 1, PMAxx (0, 5, 10, 25, 50, and 100 μM) together with Triton X-100 (5, 1, 0.1, 0.01%, and 0) were added into the samples. The mixtures were then incubated in the dark at room temperature (22–26°C) for 10 min. Subsequently, the mixtures were exposed to photolysis at different times (5, 10, 15, or 20 min) using a PMA-Lite^™^ LED photolysis device (Biotium, United States). The photolyzed samples were heat treated at 95°C for 5 min prior to DNA extraction. Extracted DNA samples were finally detected by qPCR to determine the cycle threshold (Ct) values of the mixtures. Samples without PMAxx treatment served as positive controls. Each condition was performed in triplicate.

### Quantitative real-time PCR assay

Nucleic acids of the samples were extracted using the Blood viral DNA extraction kit. The primer pairs and probes targeting the ASFV-P72 gene are listed in Table 2. The qPCR reaction system (total 20 μl) consisted of 5 μl template DNA, 10 μl 2 × reaction mix (Luna^®^ Universal Probe qPCR Master Mix, M3004S, NEB, United States), 0.4 μM forward primer, 0.4 μM reverse primer, 0.2 μM probe, and DNase free water. The qPCR reaction was performed on a Biorad CFX96 Real-Time PCR System (Bio-Rad, United States) with a denaturation step at 95°C for 1 min, followed by 45 cycles of denaturation at 95°C for 15 s and annealing/extension at 60°C for 30 s.

**Table 2:** Primers and probes used in the PMAxx-qPCR assay for detecting the P72 gene of ASFV.

Gene	Name	Sequence (5′-3′)	Amplicon size (bp)
P72	#1-Forward	TCCTGAAAGCTTATCTCTGCG	75
	#1-Reverse	AGATTGGCACAAGTTCGGAC	
	#1-Probe	FAM-TGAGTGGGCTGCATAATGGCGTT-BHQ	
P72	#2-Forward	AAGGTAATCATCATCGCACC	163
	#2-Reverse	ATCCGATCACATTACCTATTAT	
	#2-Probe	FAM-TCCGTAACTGCTCATGGTATCAATCTT-BHQ	
P72	#3-Forward	TTGATACCATGAGCAGTTACGG	189
	#3-Reverse	AGATTGGCACAAGTTCGGAC	
	#3-Probe	FAM-TGAGTGGGCTGCATAATGGCGTT-BHQ	

### Statistical analysis

The ΔCt value was used to estimate the risk and presence of infectious ASFV in tested samples. To obtain the ΔCt value, the average Ct value of a sample after PMAxx pretreatment was subtracted from the average Ct value of the same sample without PMAxx pretreatment. Resultant data was graphically presented and statistically analyzed by GraphPad Prism version 8 (GraphPad software, United States) software. A t-test was used to test the impact of variables and determine the significant differences. Ordinary one-way ANOVA test was used to analyze the significant differences of the data among different groups. A *P*-Value of *p* < 0.05 was deemed significant.

## Results

### Optimization of the PMAxx-qPCR assay

**Figure 2:**
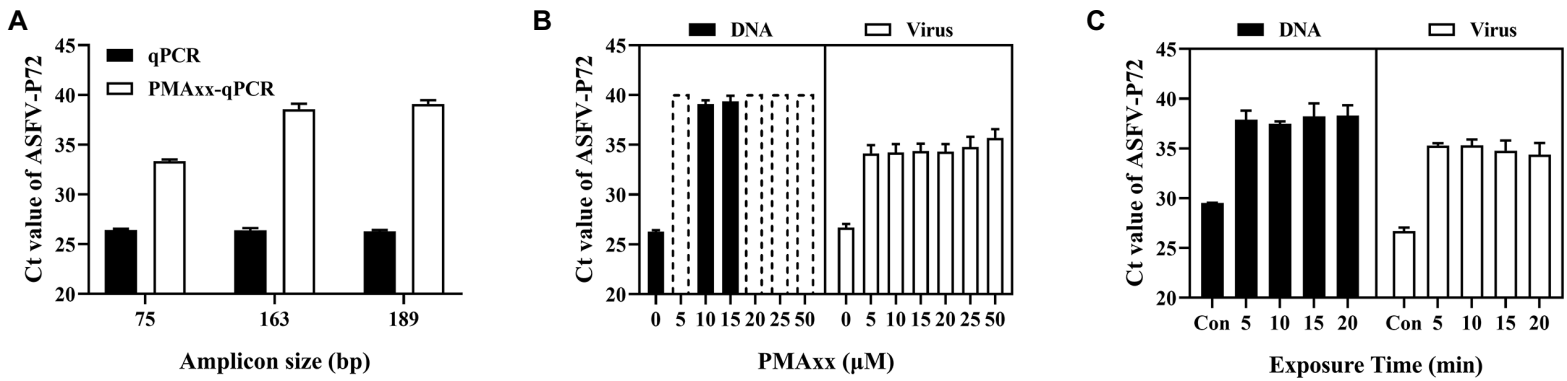
Optimization conditions of the PMAxx-qPCR assay targeting the ASFV-P72 gene for discriminating inactivated ASFV and free DNA. **(A)** Effects of different amplicon sizes on Ct values of free ASFV DNA (samples treated with 10 μM PMAxx and 15 min photolysis time). **(B)** Effects of different PMAxx concentrations on Ct values of free ASFV DNA and ASFV in 100 times PBS-diluted swine plasma inactivated at 95°C for 20 min (each concentration was exposed to a photolysis time of 15 min). **(C)** Effects of photolysis time on Ct of free ASFV DNA and ASFV in 100 times PBS-diluted swine plasma inactivated at 95°Cfor 20 min (each sample was treated with a PMAxx concentration of 25 μM). Data were shown as mean ± SD of three independent repeats. Dotted plots represent no amplification after 40 cycles and the Ct is assigned to 40 in order to calculate ΔCt.

Three main factors that may affect the ΔCt of the PMAxx-qPCR assay include the PCR amplicon size, PMAxx concentration, and photolysis time. As shown in Figure 2A, ΔCt values of the free ASFV DNA amplified using the primer/probe set #3 (amplicon size 189) were higher than those of primer/probe sets #1 (amplicon size 75) or #2 (amplicon size 163), indicating that longer amplicons were better for discrimination. Therefore, the primer/probe set #3 was used in further optimization experiments. Further tests on two types of samples (free DNA and PBS-diluted ASFV positive swine plasma) showed that PMAxx concentrations ranging from 5 μM to 100 μM (Figure 2B) and the photolysis time ranging from 5 min to 20 min (Figure 2C) had no significant difference in the ΔCt values. However, considering the fact that large amounts of nucleic acids of other organisms may be present in real life samples, a relatively high PMAxx concentration of 25 μM and longer photolysis time of 15 min was chosen for the following experiments. These conditions were also found not to have any significant interference with infectious virions when determined by cell culture as seen in Supplementary Table S1.

### Determination of heat-inactivated ASFV by the triton X-100 assisted PMAxx-qPCR assay

**Figure 3:**
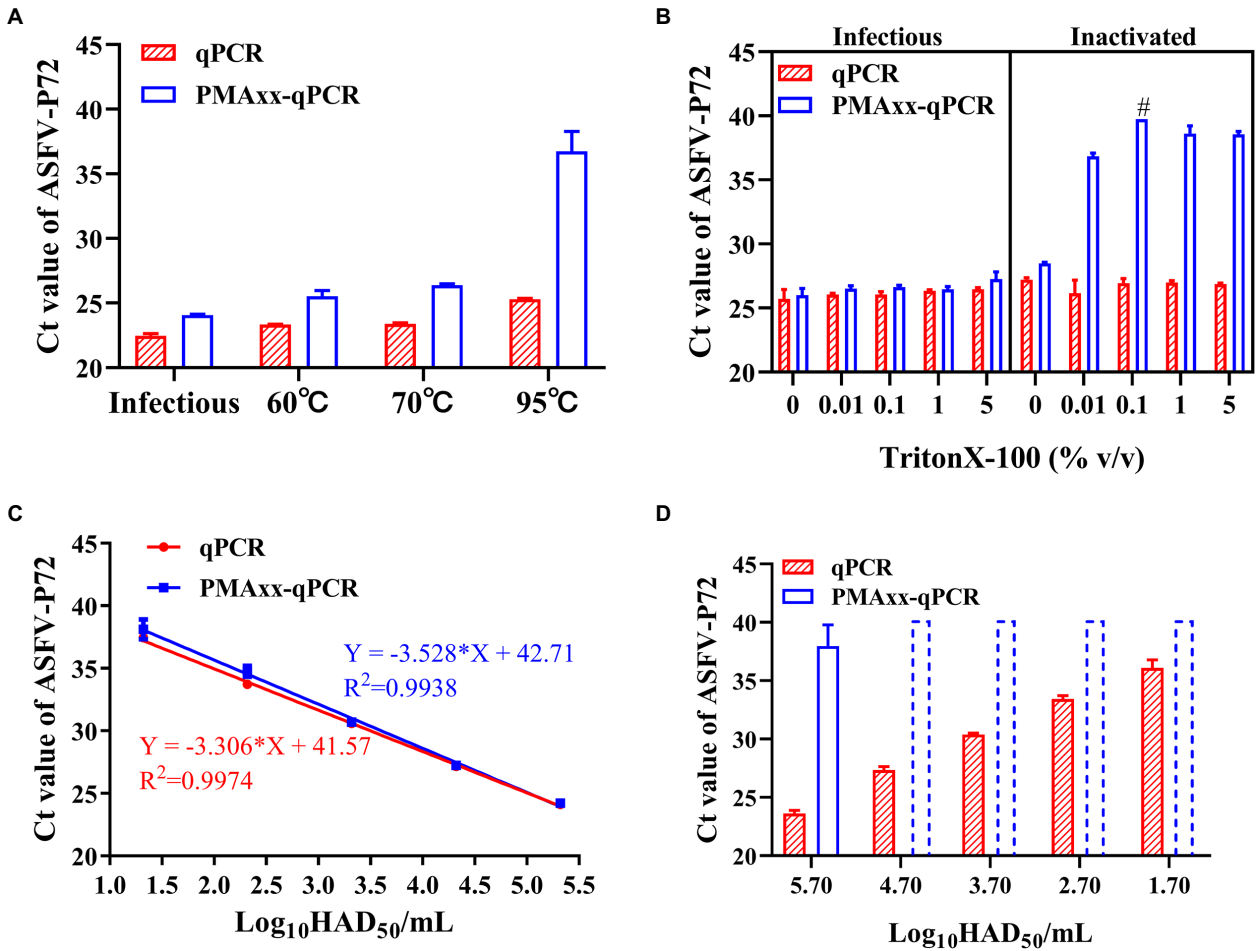
Performance of the PMAxx-qPCR assay and the Triton X-100 assisted PMAxx-qPCR assay in detecting infectious ASFV and thermo-inactivated ASFV. **(A)** Ct values of infectious ASFV and their inactivated counterparts treated at three temperatures (60°C, 70°C, or 95°C) for 20 min and determined by qPCR (without PMAxx) and PMAxx-qPCR. **(B)** Effects of the Triton X-100 assisted PMAxx-PCR assay in detecting infectious or live ASFV (left) and their counterpart inactivated ASFV at 60°C for 20 min (right). **(C)** Linear curve fitting of the qPCR and the Triton X-100 assisted PMAxx-qPCR assay using serial dilutions of infectious ASFV with 0.1% Triton X-100. **(D)** Ct values of ASFV samples inactivated at 60°C for 20 min and detected using qPCR (without PMAxx) and Triton X-100 assisted PMAxx-qPCR. Dotted plots represent no amplification after 40 cycles and the Ct is assigned to 40 in order to calculate ΔCt. Data were shown as mean ± SD of three independent repeats. #, two of the three repeats were found to have no amplification after 40 cycles.

Heat treatment is an important method of inactivating ASFV, and it has been reported that ASFV can be inactivated after heating at temperatures higher than 60°C for 20 min (Mazur-Panasiuk et al., 2019). Using this analogy, infectious ASFV samples were heat inactivated at temperatures ≥60°C, subjected to PMAxx, and results compared to their respective control samples (without heat treatment). Compared to the control, the ΔCt values were 1.6, 2.19, 3 and 11.43 for the infectious sample and samples subjected to 60°C, 70°C and 95°C temperatures, respectively (Figure 3A). These results indicated that the PMAxx-qPCR assay could optimally determine inactivated viruses heated at higher temperatures of ≥95°C, but not at mild temperatures (60°C or 70°C). The probable reason for this dismal performance at mild temperatures was thought to be related to the existence of intact ASFV capsid structures not easily broken by mild temperatures.

It has been reported that surfactants such as Triton X-100 (Coudray-Meunier et al., 2013) and SDS (Hong et al., 2021) can increase the permeability of monoazide dyes to pathogenic viruses with intact viral capsids. Therefore, we attempted to add Triton X-100 to enhance the penetration of PMAxx into the intact ASFV virions inactivated at 60°C. As shown in Figure 3B (left), Triton X-100 (5, 1, 0.1, 0.01% (v/v)) did not promote PMAxx penetration into the control (infectious ASFV viruses without heat treatment), but it increased the PMAxx penetration into ASFV samples inactivated at 60°C (Figure 3B, right). Further analysis of the ΔCt values of inactivated samples showed that 0.1% Triton X-100 had the largest ΔCt value (Figure 3B, right) and was hence chosen as an assistant to the PMAxx-qPCR assay in the following tests.

Using this concentration, further testing on serial dilutions of infectious ASFV suspensions (Figure 3C) showed that there was no significant difference between the Triton X-100-qPCR alone (without PMAxx treatment) and the Triton X-100 assisted PMAxx-qPCR. These results indicated that the Triton X-100 assisted PMAxx treatment had no effects on infectious ASFV. However, after heat treatment at 60°C for 20 min (Figure 3D), the Ct values of the qPCR did not change, but the Ct values of the Triton X-100 assisted PMAxx-qPCR increased significantly (*p* < 0.001 for all groups), showing no amplification after 40 cycles except for the highest concentration of 5.7log_10_HAD_50_/mL (Ct value: 37.96 ± 1.83).

### Application of the triton X-100 assisted PMAxx-qPCR assay

In order to verify if the Triton X-100 assisted PMAxx-qPCR assay could discriminate infectious ASFV in partially inactivated samples, a series of samples were prepared by mixing the infectious 5.32log_10_HAD_50_/mL ASFV and heat-inactivated 5.7 log_10_HAD_50_/mL ASFV at different ratios. As shown in Table 3, an increase in the percentage of infectious ASFV in the samples led to a decrease in ΔCt values. Even at 1% infectious ASFV in the samples, the ΔCt values were significantly lower than those of the ΔCt values of 100% inactivated ASFV. The same trends were observed even with lower titers of ASFV mixtures. These results demonstrated the possibility of using the Triton X-100 assisted PMAxx-qPCR assay to determine small percentages of infectious ASFV in samples by comparing the difference between the ΔCt value of the sample before inactivation and that of the same sample inactivated at 60°C for 20 min.

**Table 3:** Determining ΔCt values from mixtures of infectious and inactivated ASFV under different titers using qPCR (Ct_(-PMAxx)_) and Triton X-100 assisted PMAxx-qPCR (Ct_(+PMAxx)_) assays. A decrease in ΔCt values positively correlated to an increase in the percentage of infectious virions across all titers tested. The assay could detect as low as 1% infectious virion in the samples tested with a significantly lower ΔCt value compared to 100% inactivated ASFV (i.e., 0% infectious ASFV).

Percentage of infectious ASFV%	High titer (5.32log_10_HAD_50_/ml infectious virus mixed with 5.7log_10_HAD_50_/ml dead virus)			Middle titer (10 × dilution of high titer)			Low titer (100 × dilution of high titer)		
	Ct_(-PMAxx)_	Ct_(+PMAxx)_	ΔCt	Ct_(-PMAxx)_	Ct_(+PMAxx)_	ΔCt	Ct_(-PMAxx)_	Ct_(+PMAxx)_	ΔCt
0	23.37 ± 0.80	38.43 ± 0.76	15.06	26.50 ± 0.77	39.65^†^	>13.15	29.60 ± 0.88	NA	>10.40
1	23.12 ± 0.06	33.64 ± 0.37	10.52	27.07 ± 0.11	37.26 ± 0.91	10.19	29.95 ± 0.34	37.82^†^	>7.87
10	22.49 ± 1.26	30.30 ± 0.74	7.81	27.53 ± 0.28	34.15 ± 0.11	6.62	29.99 ± 0.30	37.42 ± 0.27	7.43
25	23.46 ± 0.21	28.75 ± 0.76	5.29	26.53 ± 1.09	32.14 ± 0.95	5.61	30.15 ± 0.11	35.54 ± 0.76	5.39
50	24.17 ± 0.23	27.85 ± 0.06	3.68	27.59 ± 0.57	31.15 ± 0.20	3.56	31.47 ± 0.85	35.43 ± 0.05	3.96
90	25.39 ± 0.21	27.27 ± 0.16	1.88	28.68 ± 0.37	30.25 ± 0.31	1.57	31.81 ± 0.37	34.43 ± 1.07	2.62
100	25.35 ± 0.27	26.31 ± 0.94	0.96	29.54 ± 0.49	30.43 ± 0.08	0.89	32.41 ± 0.27	33.85 ± 0.12	1.44

^†^two of the three repeats were found to have no amplification after 40 cycles. NA represents no amplification after 40 cycles and the Ct is assigned to 40 in order to calculate ΔCt.

### Effects of different matrices on the triton X-100 assisted PMAxx-qPCR assay

The detection of ASFV varies in different bio-matrices, this may possibly affect the performance of Triton X-100 assisted PMAxx-qPCR assays. To determine this, samples with inactivated ASFV suspended in five bio-matrices [PBS, swine blood, swine tissue homogenate, pig saliva swab (SS), and environmental swabs (ES)] were tested using both qPCR (without PMAxx treatment) and Triton X-100 assisted PMAxx-qPCR. Compared to PBS at 1× concentration, late Ct values were observed in blood and saliva matrices when detected by qPCR (Table 4). However, after adding the inactivated ASFV into 4 × or 8 × PBS-diluted matrices, early Ct values were observed. These results indicated that these two matrices would affect either the efficacy of the DNA extraction kits or contain some inhibitors that might inhibit the qPCR reaction. Additionally, blood may also affect the PMAxx treatment process since a late Ct value was observed in the undiluted blood when detected by the Triton X-100 assisted PMAxx-qPCR assay. Therefore, blood and saliva samples need to be diluted with PBS at least 4× or 8× times before detection, while tissue homogenates and the environmental swabs do not need any dilutions.

**Table 4:** Detection of inactivated ASFV suspended in different matrices by qPCR (Ct_(-PMAxx)_) and Triton X-100-PMAxx-qPCR (Ct_(+PMAxx)_) assays. Undiluted blood and saliva had later Ct values compared to PBS indicative of qPCR inhibition. These values improved after 4× and 8× dilution in PBS. Undiluted blood (1×) could also interfere with the Triton X-100-PMAxx-qPCR assay as an earlier Ct value was observed. Tissue homogenate and environmental swab had no effects on both assays.

Matrices	PBS	Dilution times of swine blood with PBS			Dilution times of pig saliva swab with PBS			Tissue homogenate	Environmental swab
Dilution	1	1	4	8	1	4	8	1	1
Ct_(-PMAxx)_	25.35 ± 0.38	27.42 ± 0.49	25.96 ± 0.62	25.64 ± 0.19	35.81 ± 0.09	29.93 ± 0.50	27.28 ± 0.25	24.01 ± 0.03	23.90 ± 0.01
Ct_(+PMAxx)_	NA	31.83 ± 0.09	NA	NA	NA	NA	NA	37.14 ± 0.83	NA

Data was shown as mean ± SD of three independent repeats. NA represents no amplification after 40 cycles.

### Evaluating the efficacy of chemical disinfectants using the triton X-100 assisted PMAxx-qPCR assay

**Figure 4:**
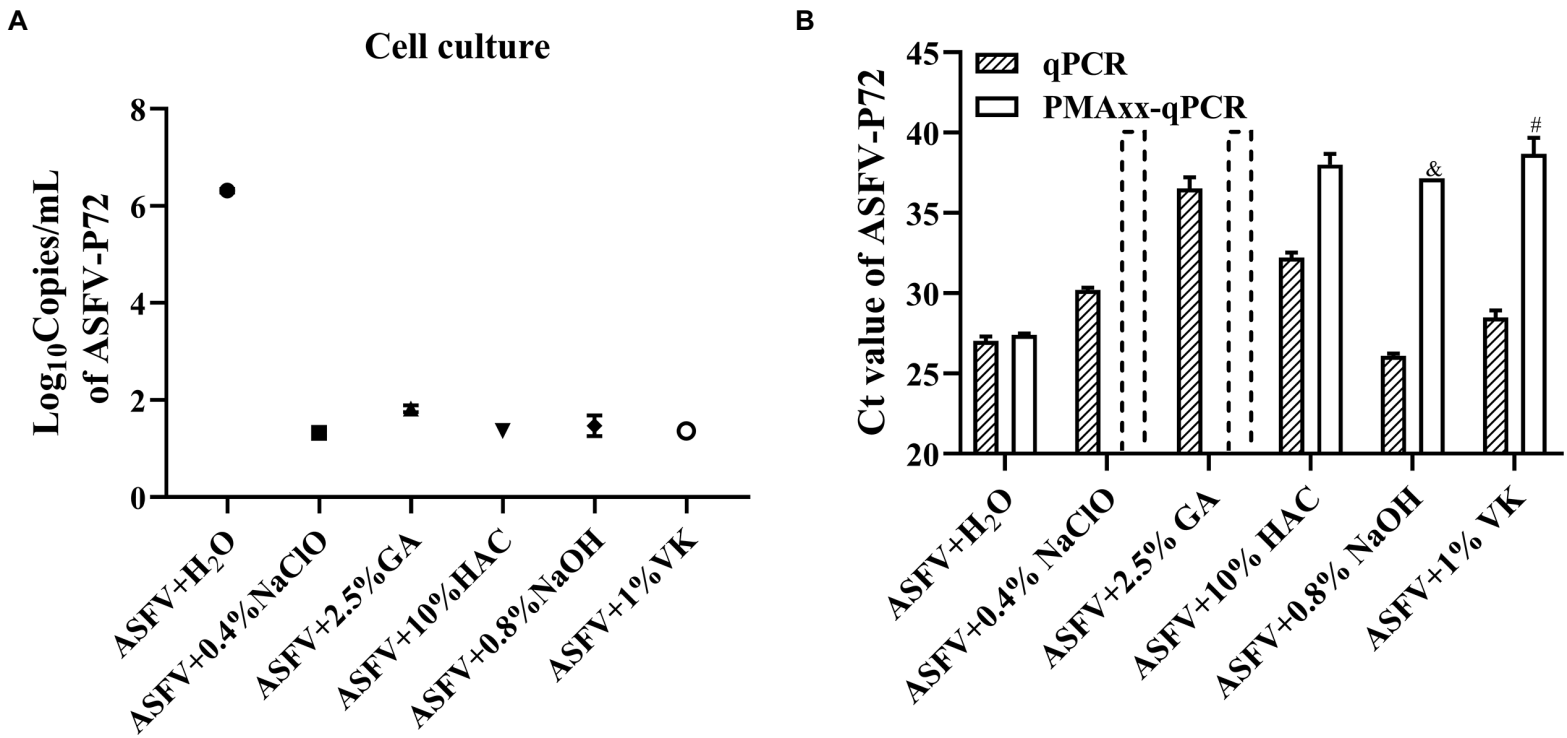
Evaluating the efficacy of the chemical disinfectants by cell culture **(A)** and the Triton X-100 assisted PMAxx-qPCR assay **(B)**. Data were shown as mean ± SD of three independent repeats. The dotted columns represent the assigned Ct value of the result of no DNA amplification after 40 cycles. &, represents two of three repeats with no amplification after 40 cycles. #, represents one of three repeats with no DNA amplification after 40 cycles.

In order to evaluate whether the Triton X-100 assisted PMAxx-qPCR assay is suitable for detecting viable ASFV after chemical disinfection, ASFV inactivated by five types of chemical disinfectants (NaClO, GA, HAc, NaOH, and VK) at different concentrations was tested, with ddH_2_O treatment serving as a positive control. Cell culture was also used to determine if there remained infectious ASFV after the disinfections. As shown in Figure 4A, the cell culture revealed that only the H_2_O-treatment group contained the infectious ASFV and no growth of ASFV could be detected after the chemical disinfections. The Triton X-100 assisted PMAxx-qPCR assay also revealed that there existed infectious ASFV in the H_2_O-treatment group because the ΔCt value was only 0.37 (*p* < 0.05). However, for the chemical disinfection groups, the Ct values of qPCR (without PMAxx treatment) alone were significantly increased except for the NaOH-treated group (Figure 4B). The increase in Ct value may be a result of the four chemical disinfectants degrading or covalently cross-linking with ASFV DNA, especially GA. After the Triton X-100 assisted PMAxx treatment, the Ct values of these chemical groups increased further to above 37 or no amplification after 40 cycles (Figure 4B). This signified that the chemicals were indeed active against ASFV.

## Discussion

It has been shown that ASFV can remain viable in natural conditions for long time periods (Arzumanyan et al., 2021) and be directly transmitted by complex transmission routes such as contact between infected and susceptible pigs (Gaudreault et al., 2020), consumption of infected pig meat (Ito et al., 2020), and bites from infected acari (*Ornithodoros* spp.; Pereira De Oliveira et al., 2020). In addition to the above primary routes, there are some potential routes for indirect transmission of ASFV through contact with virus contaminated objects and fluids such as blood, feces, urine, or saliva from infected pigs (Guinat et al., 2014; Fischer et al., 2020b; Olesen et al., 2020; Health et al., 2021). Due to there being no drugs and vaccines against ASFV, strict disinfections are the main measurements to curb the transmission of ASFV. The gold standard cell culture method is not suitable for regular monitoring of the presence of infectious ASFV in natural environments and after disinfections.

The Triton X-100 assisted PMAxx-qPCR assay developed in this study may provide some advantages over cell culture. Firstly, it could not only be used to detect ASFV DNA, but also to assess the presence of infectious ASFV in samples within 3 h. By exploring the property of PMAxx which could not penetrate the capsid of infectious virions, the PMAxx-qPCR could discriminate live virus as low as 1% from dead virus (Table 3). Secondly, it is biologically safe to perform the test without the need of a BSL-3 laboratory. By obviating the need for virus culture, the PMAxx treatment and DNA extraction can be performed within a biosafety cabinet. This makes the assay scalable with a possibility of deployment to places with limited resources, such as pig farms. Additionally, PMA assays are said to be capable of detecting live but unculturable pathogens [according to the manufacturer’s instructions, and other literature (Zhong and Zhao, 2018; Ou et al., 2021; Zhao et al., 2022)]. Lastly, considering time, and labor costs of cell culture, it is more convenient to include the PMAxx-qPCR in routine diagnosis to detect infectious ASFV.

However, validation tests need to be performed before application of the Triton X-100 assisted PMAxx-qPCR assay for real-life samples. Similar to other PMA assays (Banihashemi et al., 2012; Kragh et al., 2020; Van Holm et al., 2021), in this study, primers amplifying longer amplicons (>100 bp) for ASFV-P72 performed optimally compared to shorter amplicons. Hence chosen for further tests. Despite the advantage of using longer amplicons, no clear guideline exists towards selecting and designing amplicon lengths for optimal PMA results (Van Holm et al., 2021). As shown in Table 4, sample matrices may affect the Ct values of the qPCR assay, as well as the Triton X-100 assisted PMAxx-qPCR assay. Not only should the effects of the matrices on the DNA extraction and amplification be checked, but also on the PMAxx-DNA crosslinking. Where applicable, measures such as dilution should be used to minimize the adverse effects of the matrices. Furthermore, chemical disinfectants may affect the Ct values of the qPCR and the Triton X-100 assisted PMAxx-qPCR assay. Disinfectants recommended by the WOAH against ASFV consist of detergents, oxidizing agents, alkalis, organic acids and glutaraldehyde (Juszkiewicz et al., 2020). Among them, NaClO, GA, HAc, NaOH, and VK are widely used to sanitize contaminated agricultural and veterinary facilities, especially in the farm settings (Turner and Williams, 1999; Kalmar et al., 2018). However, different disinfectants may degrade DNA or covalently cross-link with ASFV DNA during disinfection, resulting in increased Ct values (Figure 4B). All these factors should be validated first to make sure that the PMAxx-qPCR assay can accurately discriminate infectious ASFV in samples.

In summary, a Triton X-100 assisted PMAxx-qPCR assay was developed to discriminate infectious ASFV from inactivated ASFV based on changes in Ct value (ΔCt). Under optimum conditions, the limit of detection of the PMAxx-qPCR assay was 2.32log_10_HAD_50_/mL of infectious ASFV. Testing different samples showed that the PMAxx-qPCR assay was effective in evaluating intact ASFV virions after treatment by heat or chemical disinfectants within 3 h. However, validation should be performed first to determine the ΔCt cutoff value for assessing the presence of infectious ASFV in different types of samples.

## Data availability statement

The original contributions presented in the study are included in the article/Supplementary material, further inquiries can be directed to the corresponding authors.

## Author contributions

HW and JY conceived and designed the project and revised the manuscript. HL and FM performed the experiments and wrote the original manuscript. RN made suggestions in this study and revised the manuscript. MJ and FM provided the ASFV viruses. DL was responsible for sample collection. RN, WH, PH, WZ, and DB performed data analyses. All authors contributed to the article and approved the submitted version.

## Funding

This work was supported by Key technologies for ASFV control from Department of Science and Technology of Hubei province, China (Grant No: 2019ABA08) and the National Key Research and Development Program of China from the Ministry of Science and Technology of China (No. 2018YFC0840402).

## Conflict of interest

The authors declare that the research was conducted in the absence of any commercial or financial relationships that could be construed as a potential conflict of interest.

## Publisher’s note

All claims expressed in this article are solely those of the authors and do not necessarily represent those of their affiliated organizations, or those of the publisher, the editors and the reviewers. Any product that may be evaluated in this article, or claim that may be made by its manufacturer, is not guaranteed or endorsed by the publisher.
