# Supplementary material for: A triton X-100 assisted PMAxx-qPCR assay for rapid assessment of infectious African swine fever virus
Source: Front Microbiol. 2022 Dec 5;13:1062544. doi: 10.3389/fmicb.2022.1062544 (PMC9760672; doi:10.3389/fmicb.2022.1062544)
Supplement: Supplementary file 1 [file Table_1.docx]

TABLE S1. The effects of the pre-treatments of 25 μM PMAxx, 0.1% Triton X-100 and 15 min photoactivation on viral infectivity by cell-culture.

| Groups | Titers of ASFV (log_10_HAD_50_/mL) |
| --- | --- |
| ASFV without pre-treatment | 3.35±0.25 |
| ASFV with 0.1% Triton X-100 pre-treatment | 3.38±0.19 |
| ASFV with 25 μM PMAxx and 15 min photoactivation | 3.23±0.55 |
| ASFV with pre-treatments of 0.1% Triton X-100, 25 μM PMAxx and 15 min photoactivation | 3.06±0.21 |

Data was shown as mean ± SD of three independent repeats (*p*=0.65).
